# Nutrient Inputs Stimulate Mercury Methylation by Syntrophs in a Subarctic Peatland

**DOI:** 10.3389/fmicb.2021.741523

**Published:** 2021-10-04

**Authors:** Spencer Roth, Brett A. Poulin, Zofia Baumann, Xiao Liu, Lin Zhang, David P. Krabbenhoft, Mark E. Hines, Jeffra K. Schaefer, Tamar Barkay

**Affiliations:** ^1^Department of Environmental Sciences, Rutgers, The State University of New Jersey, New Brunswick, NJ, United States; ^2^Department of Biochemistry and Microbiology, Rutgers, The State University of New Jersey, New Brunswick, NJ, United States; ^3^Department of Environmental Toxicology, University of California, Davis, Davis, CA, United States; ^4^Department of Marine Sciences, University of Connecticut, Groton, CT, United States; ^5^Department of Biological Sciences, University of Massachusetts, Lowell, MA, United States; ^6^Department of Physical and Environmental Sciences, Texas A&M University - Corpus Christi, Corpus Christi, TX, United States; ^7^United States Geological Survey, Upper Midwest Water Science Center, Mercury Research Laboratory, Middleton, WI, United States

**Keywords:** climate change, peatland, *hgcA*, syntrophy, mercury methylation

## Abstract

Climate change dramatically impacts Arctic and subarctic regions, inducing shifts in wetland nutrient regimes as a consequence of thawing permafrost. Altered hydrological regimes may drive changes in the dynamics of microbial mercury (Hg) methylation and bioavailability. Important knowledge gaps remain on the contribution of specific microbial groups to methylmercury (MeHg) production in wetlands of various trophic status. Here, we measured aqueous chemistry, potential methylation rates (k_*meth*_), volatile fatty acid (VFA) dynamics in peat-soil incubations, and genetic potential for Hg methylation across a groundwater-driven nutrient gradient in an interior Alaskan fen. We tested the hypotheses that (1) nutrient inputs will result in increased methylation potentials, and (2) syntrophic interactions contribute to methylation in subarctic wetlands. We observed that concentrations of nutrients, total Hg, and MeHg, abundance of *hgcA* genes, and rates of methylation in peat incubations (k_*meth*_) were highest near the groundwater input and declined downgradient. *hgcA* sequences near the input were closely related to those from sulfate-reducing bacteria (SRB), methanogens, and syntrophs. Hg methylation in peat incubations collected near the input source (FPF2) were impacted by the addition of sulfate and some metabolic inhibitors while those down-gradient (FPF5) were not. Sulfate amendment to FPF2 incubations had higher k_*meth*_ relative to unamended controls despite no effect on k_*meth*_ from addition of the sulfate reduction inhibitor molybdate. The addition of the methanogenic inhibitor BES (25 mM) led to the accumulation of VFAs, but unlike molybdate, it did not affect Hg methylation rates. Rather, the concurrent additions of BES and molybdate significantly decreased k_*meth*_, suggesting a role for interactions between SRB and methanogens in Hg methylation. The reduction in k_*meth*_ with combined addition of BES and molybdate, and accumulation of VFA in peat incubations containing BES, and a high abundance of syntroph-related *hgcA* sequences in peat metagenomes provide evidence for MeHg production by microorganisms growing in syntrophy. Collectively the results suggest that wetland nutrient regimes influence the activity of Hg methylating microorganisms and, consequently, Hg methylation rates. Our results provide key information about microbial Hg methylation and methylating communities under nutrient conditions that are expected to become more common as permafrost soils thaw.

## Introduction

Methylmercury (MeHg) is a potent neurotoxic compound that bioaccumulates and biomagnifies in aquatic food webs (USEPA, 1997). Northern freshwater wetlands are ecosystems that have been shown to be net sources of MeHg (Loseto et al., 2004; Tjerngren et al., 2012), and elevated MeHg concentrations in fish and mammals have been reported in the Arctic and subarctic regions (Dietz et al., 2013). Northern wetlands receive inorganic mercury primarily through atmospheric deposition of Hg(II) and direct uptake of gaseous elemental mercury by plants [and subsequent oxidation to Hg(II)] (Enrico et al., 2016; Jiskra et al., 2017; Obrist et al., 2017). Under anoxic conditions in wetlands, Hg(II) can be methylated to form MeHg (Loseto et al., 2004; Mitchell et al., 2008; Tjerngren et al., 2012; Gordon et al., 2016; Schaefer J. K. et al., 2020).

Mercury methylation is an anaerobic process carried out by bacteria and archaea. Formerly, the methylation of Hg has been largely attributed to sulfate- and iron-reducing bacteria (SRB and IRB, respectively) (Gilmour and Henry, 1992; Fleming et al., 2006). More recently, the discovery of the *hgcAB* genes encoding for the Hg methylase and associated ferredoxin (Parks et al., 2013) has revealed that several additional guilds of anaerobic microorganisms can methylate Hg, including methanogens (Gilmour et al., 2013, 2018; Yu et al., 2013, 2018), fermenters (Gilmour et al., 2013) and syntrophs (Gilmour et al., 2013; Yu et al., 2018). This advancement has provided an important gene marker for identifying environments and microbial taxa with a potential for Hg methylation (Bae et al., 2014; Schaefer J. K. et al., 2014; Christensen et al., 2016; Jones et al., 2020; McDaniel et al., 2020; Peterson et al., 2020). However, the contribution of newly identified methylating guilds to MeHg production in the environment remains to be demonstrated.

In peat-forming wetlands, primary production exceeds decomposition, resulting in the buildup of organic carbon stores (Gorham, 1991; Charman et al., 2013; Nichols and Peteet, 2019). Carbon degradation in the anoxic zone occurs through a combination of primary and secondary fermentation processes and anaerobic respiration. The relative contribution of these different pathways depends upon the availability of nutrients and suitable electron acceptors. When terminal electron acceptors are scarce, mineralization of fermentation products is driven primarily through syntrophy between secondary fermenters and methanogens (McInerney et al., 2008, 2009; Schmidt et al., 2016), whereby substrates are metabolized by two groups of organisms under conditions that are not thermodynamically favorable for either of them alone (Morris et al., 2013). Notably, many Hg methylating organisms are within metabolic guilds involved in anaerobic carbon degradation in peat-forming wetlands. The contribution of these metabolic guilds to MeHg formation and influence of nutrient availability on Hg methylating communities remain to be understood.

Permafrost loss in the Arctic and subarctic causes hydrological changes (Box et al., 2019) that can contribute nutrients in the form of terminal electron acceptors to wetland ecosystems (Reyes and Lougheed, 2015; Schadel et al., 2016). In addition, changes in wetland hydrology has the potential to contribute previously sequestered Hg to wetland active layers (Rydberg et al., 2010; Ci et al., 2020; Mu et al., 2020; Schaefer K. et al., 2020). Several studies have linked Hg methylation potential and MeHg accumulation to wetland trophic status (Tjerngren et al., 2012; Gordon et al., 2016; Poulin et al., 2019; Schaefer J. K. et al., 2020), but few have investigated the microbial processes driving these relationships, such as impacts of nutrient inputs on microbial community dynamics and corresponding Hg methylation potentials. Forecasting the implications of a warming climate on Hg risk to northern ecosystems necessitates an improved understanding of Hg methylating communities and methylation potential across nutrient regimes.

Here, we tested the hypothesis that nutrient inputs influence microbial community structures, increasing Hg methylation rates and MeHg accumulation in a fen wetland in interior Alaska. We characterized the biogeochemical conditions of the wetland and the active peat microbiome through 16S rRNA sequencing, *hgcA* amplicon sequencing, and metagenomics at six sites across a nutrient gradient. Potential rates of Hg methylation were measured along the gradient in incubations aimed at stimulating or inhibiting specific microbial guilds, to ultimately elucidate the metabolic pathways involved in MeHg formation. In this study, we show how inputs of nutrients can promote Hg methylation in subarctic peatlands by stimulating respiratory pathways when electron acceptors are present (e.g., sulfate) and through syntrophic interactions when these electron acceptors become depleted.

## Materials and Methods

### Site Description and Sampling

Six sites spanning a distance of 73 m within a fen wetland, referred to as Frozen Pond Fen (FPF) were identified in interior Alaska near Fairbanks (N64.91417, W147.83487). A strong geochemical gradient was observed across the fen, and Poulin et al. (2019) identified evidence for a groundwater input near FPF1. A site map is presented in Supplementary Figure 1, and site coordinates and pore water geochemistry are summarized in Table 1. Average monthly precipitation and daily temperature were collected from NOAA National Centers for Environmental Information station USC00503368 and are presented in SI. Average daily high temperature was similar between 2016 and 2018 (Supplementary Figure 2), however, average monthly rainfall prior to sampling dates was higher in 2016 (Supplementary Figure 3). Pore water and peat samples were collected in summers of 2016 (July 13) and 2018 (June 27). Samples for molecular characterization of the soil microbial communities were collected from 5 cm below the water table at each site. Approximately 1 g of soil was collected with 75% ethanol rinsed forceps and placed into 3 mL LifeGuard Soil Preservation Solution (Qiagen). Samples were stored on ice and transferred for temporary storage (−20°C) prior to shipping to Rutgers University, NJ, (within 5 days) where they were stored at −80°C until processing.

**TABLE 1:** Sampling locations in Frozen Pond Fen (2016 and 2018) and filtered pore water geochemical data from 10 cm below the water table (2016).

Site	FPF1	FPF2	FPF3	FPF4	FPF5	FPF6
Latitude	64.914417	64.914233	64.9142	64.914167	64.91415	64.91415
Longitude	−147.83545	−147.8351	−147.8349	−147.8349	−147.8344	−147.834
Distance from FPF1 (m)	0	26.8	35.4	39.1	57.0	73.1
C:N Average ± SD	24.5 ± 0.1	18.7 ± 1.1	24.7 ± 3.0	24.5 ± 6.0	21.6 ± 1.6	25.4 ± 2.6
Ca^2+^ (mmol/L)	2.18	1.18	0.31	0.33	0.19	0.07
pH	6.84	6.57	5.94	6.03	5.28	5.05
Conductivity (μS/cm)	1280	702	202	193	119	40
DOC (mg C/L)	271	109	57.2	49.3	34.3	28
SUVA_254_ (L/mg C m)	2.6	2.8	3.2	3.3	3.1	3.2
SO_4_^2–^ (μmol/L)	38	BD^1^	BD	BD	BD	BD
S(-II) (μmol/L)	1.6	0.6	0.4	BD	BD	BD
Total Fe (μmol/L)	103	1.9	2.7	4	13	10.9
%Fe(II)	90%	BD	100%	100%	99%	100%
Mn (μmol/L)	15.6	12.5	5.4	BD	2.5	2.0
Acetate (mg C/L)	0.1	1.3	1.8	0.4	0.3	0.3
Hg(0)_(aq)_ (pg/L)	151.4	24.5	107.3	23	31.7	21.1
Total Hg (ng/L)	7.6	4.1	4.3	2.3	3.2	2.4
MeHg (ng/L)	1.37	0.13	0.22	0.08	0.05	0.05

*^1^BD, below detection.*

*Detection limits were 1.0 μmol/L sulfate, 0.3 μmol/L sulfide, 0.2 μmol/L Fe(II), and 1 μmol/L Mn (Poulin et al., 2019).*

Peat soil samples for Hg methylation assays were collected from the saturated zone above the underlaying permafrost (∼5–10 cm below the water table) and placed in 0.95 L soda-lime glass Mason jars which were completely filled and immediately sealed. Peat soils were stored on ice for approximately 6 h prior to the initiation of methylation assays.

### Pore Water Biogeochemical Parameters

Sampling procedures, sample processing, storage, and analyses in 2016 were performed as previously described (Poulin et al., 2019) and included characterization of pore water pH, conductivity, anions, cations, DOC, specific UV absorbance at 254 nm (SUVA_254_), and Hg species including total Hg (THg), MeHg, and dissolved gaseous mercury [Hg(0)_(aq__)_]. In 2018, pH was measured *in situ.* Pore water was collected from ∼5 cm below the water table into 15 mL Falcon tubes for cation and anion analysis, and into pre-muffled amber glass vials for DOC analysis. All samples were stored on ice for ∼6 h before being frozen (ions) or kept at 4°C (DOC) and shipped to Rutgers University for analysis.

DOC was measured in duplicate for each site using a Shimadzu TOC-VCSH. The lowest standard of 1 ppm represented the limit of detection. Ions were measured by ion chromatography (Thermo Scientific Dionex Aquion Ion Chromatograph) at the Dawson Lab in the Department of Environmental Sciences at Rutgers University. Anions were eluted on a Dionex IonPac AS11 4 × 50 mm guard column and 4 × 250 mm column coupled to a Dionex AERS 500 carbonate anion suppressor with a 9 mM NaCO3 eluent over 30 min. Cations were eluted on a Dionex IonPac CS12a 4 × 40 mm guard column and a 4 × 250 mm column coupled to a Dionex SC-CERS 500 salt converter cation suppressor with 20 mM methanesulfonic acid eluent over 20 min.

Detection limits are reported in Supplementary Table 2. Peat was homogenized and collected in silver foil for C:N analysis. Total organic carbon and total nitrogen were measured with a Carlo Erba NA1500 series 2 elemental analyzer in the Rutgers Department of Marine and Coastal Sciences.

### Mercury Methylation Assays

Potential methylation assays were determined on the day of sampling using two different types of incubations. To account for variation in pore water chemistry and Hg speciation and the impact they may have on the availability of the substrate to the methylating communities (Gilmour et al., 1998), ^198^HgCl_2_ (for experiments performed in 2016) or ^200^HgCl_2_ (for experiments performed in 2018) was preequilibrated with pore water from FPF2 prior to its addition to incubations from both sites. Incubations were conducted in sites FPF2 and FPF5, as geochemical parameters in FPF1 were drastically higher than the rest of the gradient, and many parameters were considerably low or below detection in FPF4. Peat from FPF2 and FPF5 was initially homogenized by hand. Incubations were prepared in duplicate or triplicate with 5 g saturated peat added to 20 mL serum bottles under N_2_ flow. Individual samples were prepared for initial time point (*T* = 0 h) and final time point (*T* = 24 h) for each treatment. Each peat incubation was amended by pipette with the appropriate substrate/inhibitor (see below) and an enriched Hg(II) isotope (^198^Hg or ^200^Hg) pre-equilibrated with FPF2 site porewater for 4 h. Incubations were mixed following amendment addition with a borosilicate glass stir rod, sealed with Teflon stoppers, and flushed with N_2_. Time 0 incubations were immediately stored at −20°C and incubations were carried out at room temperature for 24 h, followed by storage at −20°C that stopped the experiment. Experimental treatments included a control (no amendment), sulfate addition (∼0.11 mM sodium sulfate), molybdate addition (∼0.2 mM sodium molybdate), 2-bromoethanesulfonic acid addition (∼25 mM BES), and a combination of both molybdate and BES (∼0.2 mM sodium molybdate, ∼25 mM BES). ^198^HgCl_2_ was incubated with pore water from site FPF2 for 4 h prior to spiking incubations. Molybdate, a structural analog to sulfate, has been shown to inhibit ATP sulfurylase (Peck, 1959; Taylor and Oremland, 1979) and is a specific inhibitor of dissimilatory sulfate reduction (Biswas et al., 2009; Sela-Adler et al., 2017). Molybdate concentrations used in this study (0.2 mM) have previously shown near complete inhibition of sulfate reduction in freshwater sediment with <0.1 mM sulfate (Fleming et al., 2006). BES is an analog of coenzyme M (Gunsalus et al., 1978) and specific inhibitor of methanogenesis (Oremland and Polcin, 1982; Compeau and Bartha, 1985).

Details regarding the analysis of enriched mercury stable isotopes in the incubations are presented in Supplementary Material. Peat was freeze-dried prior to Hg analysis. Potential Hg methylation rate constants (k_*meth*_) in 2016 were calculated as [Me^198^Hg]/[T^198^Hg] × 100% (Note: T^198^Hg is total ^198^Hg detected in dried peat). Time 0 incubation k_*meth*_ averages were subtracted from time final incubation k_*meth*_ after 24 h. Due to limitations of sample material in 2018, T^200^Hg was not analyzed in each incubation and therefore, an average T^200^Hg was calculated for each site and used to calculate k_*meth*_ as [Me^200^Hg]/[T^200^Hg] × 100%.

### Peat Slurry Incubations to Quantify Volatile Fatty Acid Dynamics

In the laboratory, slurries from each site were prepared using previously established procedures (Duddleston et al., 2002; Zhang et al., 2020) in a glove bag filled with 100% N_2_ gas. Excess pore water was squeezed from the peat and woody plant roots and any remaining green vegetation were removed. About 100–300 g peat (wet weight) was homogenized with pore water in a blender at a ratio of ∼1:3 wet weight peat: total volume slurry. Slurries prepared in this manner contained 0.04–0.08 g dried peat per mL. In each of the 200 mL serum bottles, 50 mL of slurries were mixed with 100 mL MilliQ water and incubated for 50 days. On day 1, one set of serum bottle (*n* = 2) per treatment was amended with 1 mL of 500 mM BES to inhibit methanogenesis (Compeau and Bartha, 1985). Slurry samples (1.0 mL) for anion analysis were periodically collected from incubation bottles using a plastic 1.0 mL syringe. Samples were centrifuged (12,000 × *g*) for 5 min and the supernatant filtered through a 0.22 μm sterilized PES syringe filter (Celltreat). Anions (acetate, propionate, butyrate, SO_4_^2–^, and NO_3_^–^) were measured by injecting 25 μL of filtered supernatant into an automated Dionex DX600 ion chromatograph (Thermo Scientific) fitted with an AS11HC ion exchange column (4 mm) with suppression and with a potassium hydroxide gradient eluent generator and conductivity detector.

### Nucleic Acid Extraction and Sequencing

Frozen peat samples were thawed at 4°C and centrifuged at 2500 × *g* for 5 min to remove the Lifeguard storage solution. RNeasy Powersoil Total RNA kit (Qiagen) and DNA extraction kit (Qiagen) were used to extract RNA and DNA, respectively, from the same original sample and as described by the manufacturer. RNA and DNA were quantified using both Qubit (ThermoFisher) and Nanodrop (ThermoFisher). RNA integrity was determined through gel electrophoresis and RNA and DNA extracts were stored at −80°C and −20°C, respectively. To prepare cDNA, RNA extracts were DNase treated using Turbo DNase Free Kit (Sigma) and Supercript III First Strand cDNA Kit (ThermoFisher). The V4 region of the 16S rRNA genes was amplified using primers 515F and 806R (Apprill et al., 2015; Parada et al., 2016) from DNA extracts and cDNA preparations, and amplicons were sequenced by Illumina MiSeq at MRDNA (Shallowater, TX) (2016 libraries) and at the Rutgers Genome Cooperative (2018 libraries).

*hgcA* amplicon libraries were prepared for DNA extracts from FPF1-6 (2016) with primers and protocol developed by Schaefer J. K. et al. (2014). Amplification products were separated by gel electrophoresis and bands corresponding to 650 bp were excised and purified (Wizard, Promega). Purified PCR products were shipped to MRDNA for sequencing by the PacBio Sequel platform according to manufacturer’s protocol, including the CCS2 algorithm which was used to generate a consensus sequence as secondary analysis.

DNA extracts from sites FPF1-6 (2016 and 2018) were used for shotgun metagenomic analysis by the Community Science Program of the Department of Energy Joint Genome Institute (CSP 504041). Details on rRNA removal, library construction, sequencing, and downstream analysis can be found on the JGI genome portal.^1^

### Amplicon Sequence Analysis

Resulting raw sequence files were processed in QIIME2 (version 2018.6). Briefly, samples were imported, demultiplexed, pair-joined, trimmed of adapters, and denoised using the Deblur plugin (Amir et al., 2017). A phylogenetic tree was constructed with Fasttree (Price et al., 2010) and diversity metrics were calculated using the Diversity plugin. Samples were rarified to 8122, a depth which retained most samples and features. Taxonomic assignment was done using a Baysean-Naïve classifier trained on the V4 region of the Silva database (132–99%) (Quast et al., 2013; Bokulich et al., 2018). Biological replicates were averaged for all sites.

*hgcA* consensus amplicon sequences were demultiplexed and primer sequences were removed. Reference *hgcA* sequences based on ORNL predicted methylators list (Gionfriddo et al., 2019) were used to train and test a naïve-Bayesian classifier (Pedregosa et al., 2011) using the QIIME2 framework. Amplicon sequences were classified, and family level taxonomy is presented.

### Metagenomic Analysis

Shotgun metagenomes were processed through the JGI IMG pipeline (v 5.0.0) (Chen et al., 2019). Translated protein sequences were downloaded from the JGI Genome portal and a Hidden Markov Model presented in Gionfriddo et al. (2020) was used to search metagenome assemblies for HgcA sequences with an inclusion threshold of *e*-value <8 × 10^–10^. To determine this *e*-value threshold for including a sequence in our analysis, we used 627 reference sequences for Pfam03599 which contains CO dehydrogenase/acetyl-CoA synthase delta subunit. We removed Pfam03599 sequences containing the HgcA cap region [N(V/I)WCAA] (Smith et al., 2015) and ran the remaining 535 sequences against an HgcA HMM model using hmmsearch in HMMER.^2^ All sequences with *e*-value <1 × 10^–5^ were manually checked for the HgcA-like cap region following an NCBI BLAST search. An *e*-value threshold of <8 × 10^10^ was determined to exclude non-HgcA CO dehydrogenase/acetyl-CoA synthase delta subunit sequences. The *e*-value <8 × 10^–10^ cutoff includes long sequences and sequences that were downstream of the NVWCAA cap with confidence that they were incomplete HgcA. Corresponding DNA contigs were selected and taxonomic annotation assigned through the JGI IMG 5.0.0 pipeline for *hgcA* validated sequences was obtained from the JGI Genome portal. Because taxonomic affiliation was assigned on the entire contig (not just *hgcA*), greater resolution was obtained when compared to a tree placement method (i.e., pplacer). Final relative abundance of *hgcA* from each site was calculated as sequences that passed HMM filtering times the scaffold/contig coverage. Abundances were normalized per 10^6^ total genes. We report *hgcA* taxonomy at the class level for all homologs and at the family level taxonomy for hits >60% identity score.

Marker genes for microbial metabolism were selected from the annotated metagenomes on JGI IMG database. KEGG Orthology ID K00399 was used to identify *mcrA* genes, K11181 was used to identify *dsrB* genes, and K00520 for *merA* genes. Since the relative abundances of *dsrA* and *dsrB*, encoding the alpha and beta subunits of the dissimilatory sulfite reductase, respectively, were highly correlated across the wetland, we focus our results and discussion on the latter, *dsrB* (K11181).

### Statistics and Data Distribution

Shotgun metagenomes are available on the DOE JGI Genome Portal (see text foonote 1) and IMG platform.^3^ 16S rRNA sequences are deposited at the National Science Foundation Arctic Data Center (Barkay and Roth, 2019). Statistical analyses were conducted in Microsoft Excel and R using the *vegan* (Oksanen et al., 2020) and *stats* packages. Correlations were conducted in R using the *stat_cor* function in *ggpubr* package. Distance across the fen was used as the independent variable for correlation analyses unless otherwise noted. All data used in coorelation analyses were tested for normality using a Shapiro-Wilk test. Normal data were subjected to Pearson coorelation and Spearman correlations were calculated for non-normal data. Methylation incubation data were determined to be normally distributed through Shapiro-Wilk test. One-way ANOVA was used to determine significance followed by *t*-test to determine significance between treatments.

## Results

### Site Description

Six sites were chosen in Frozen Pond Fen (FPF) along a natural groundwater-driven geochemical gradient. Lateral gradients of all measured parameters were apparent between FPF1 (nearest the groundwater source) and FPF6 (73 m from FPF1) (Table 1 and Supplementary Figure 4). Conductivity and Ca^2+^ were highest at FPF1 and significantly declined across the gradient (Pearson’s *r* = -0.903, *p* = 0.008 and Pearson’s *r* = −0.899, *p* = 0.009, respectively) (Table 1 and Supplementary Figure 4), indicative of a groundwater source near FPF1. pH also significantly declined along the fen gradient from site 1 to site 6 (Pearson’s *r* = −0.966, *p* < 0.001) with a maxima of pH 6.8 at FPF1. Dissolved organic carbon (DOC) decreased significantly from FPF1 to FPF6 (Spearman’s ρ = −1, *p* = 0.003), with reversed trends in the DOM SUVA_254_ (Pearson’s *r* = 0.828, *p* = 0.042), suggesting a lower aromaticity of the DOC nearest the source. The concentrations of sulfate and sulfide were highest in FPF1 (38 and 1.6 μmol/L, respectively), both declining to <1 μmol/L at FPF2 (27 m from FPF1). Manganese and iron concentrations were highest nearest FPF1 (15.6 and 103 μmol/L) but declined rapidly to near or below detection with distance from the groundwater source. *Calliergon sp., Scorpidium revolvens*, and *Carex aquatalis* were the dominant plant species in the Frozen Pond Fen with little variation between sites (Poulin et al., 2019). No correlation was observed between C:N and distance from FPF1, indicating that no large-scale differences in wetland trophic status occur across the fen.

As for most other parameters, pore water concentrations of dissolved gaseous mercury [Hg(0)_(aq__)_], dissolved total mercury, and dissolved MeHg declined significantly with increasing distance from FPF1 (THg Pearson’s *r* = −0.854, *p* = 0.02; MeHg Spearman’s ρ = −0.928, *p* = 0.008) (Table 1 and Supplementary Figure 5). A positive correlation between MeHg and total dissolved Hg concentrations was observed (Spearman’s ρ = 0.81, *p* = 0.049) across all six sites. These results correspond to a decrease in the percentage of Hg as MeHg (%MeHg) along the gradient, with MeHg accounting for 18% of total Hg at FPF1 and for only 2% in FPF5 and FPF6. Interestingly, all measured Hg species were elevated in site FPF3 relative to those in FPF2 with Hg(0)_(aq)_ and MeHg more than 4-fold and 1.7-fold higher, respectively, despite a minimal increase in total Hg.

The FPF sites were resampled in 2018, 2 years after the initial analyses. Resampling was conducted to validate previous gradient trends and to determine how the fen changed over the course of 2 years. Significant negative trends in pore water pH, Ca^2+^, and DOC were observed across the fen again in 2018 (Supplementary Figure 4 and Supplementary Table 2). Average pH and sulfate (FPF1 only) was higher in 2018 when compared to 2016 (2018 pH = 6.25, 2016 pH = 5.95) (Supplementary Figure 4 and Supplementary Table 4). Rainfall was higher in 2016 than 2018 immediately preceding sampling which may have contributed to some of the differences in porewater chemistry between sampling years (Supplementary Figure 3). *In situ* Hg species were not measured in 2018.

### Microbial Community

The microbial community was assessed through (1) 16S rRNA gene and transcript metataxonomic analysis, (2) shotgun metagenomic analyses, and (3) high-throughput amplicon sequencing of *hgcA* genes (2016 only). This multifaceted approach allowed us to identify shifts in the microbial community composition between sites, identify potential methylators, and compare sequence-based approaches.

**FIGURE 1:**
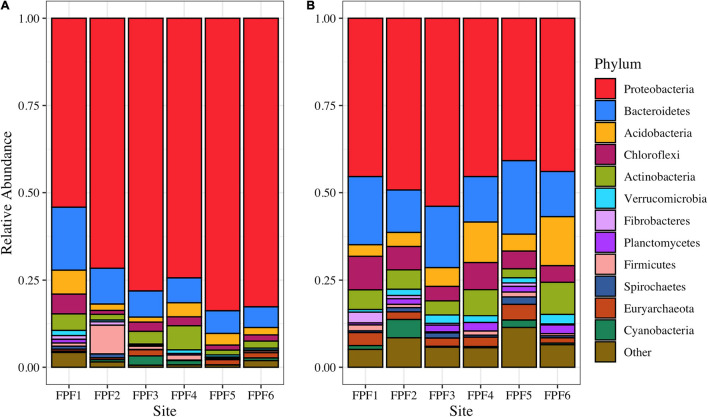
Microbial community structure based on 16S rRNA amplicon reads in Frozen Pond Fen in **(A)** 2016 and **(B)** 2018. Phylum level taxonomy is displayed, and the top 12 phyla are presented. Phyla with relative abundances <2% are grouped into “Other”.

The microbial community as assessed by 16S rRNA sequencing of cDNA was dominated by *Proteobacteria* across all sites (Figure 1). Site FPF1 had the highest community diversity (Supplementary Table 3), and the relative abundance of *Proteobacteria* increased from the nutrient rich to nutrient poor locations of the fen. In 2018, the community composition and diversity were less variable across the gradient, but overall community alpha diversity was significantly higher when compared to 2016 (Kruskal-Wallis, *p* = 0.0039) (Supplementary Table 3). Several families with known methylating organisms were identified in the 16S rRNA sequencing libraries (Supplementary Table 4), including *Syntrophaceae, Geobacteraceae, Desulfobulbaceae, Desulfobacteraceae, Ruminococcaceae*, and *Methanoregulaceae.*

**FIGURE 2:**
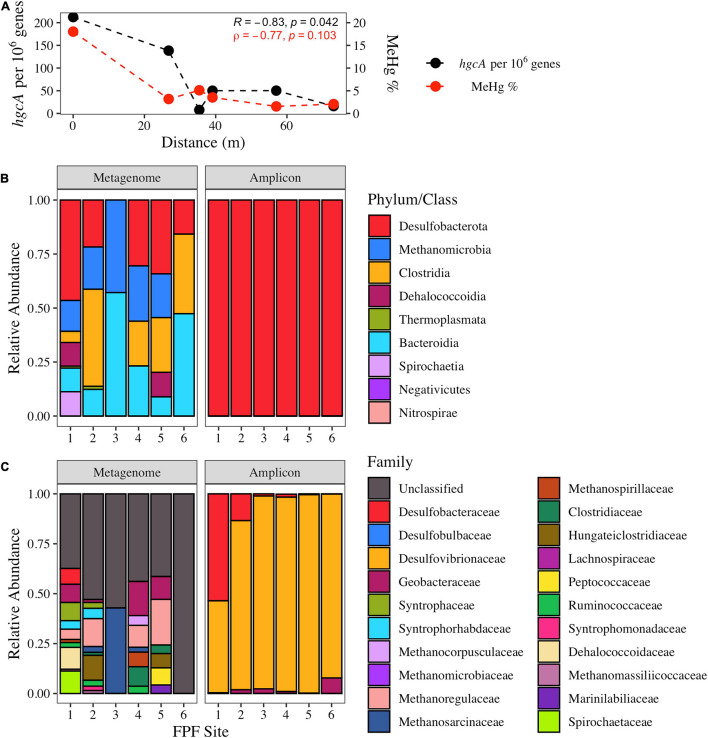
The abundance and taxonomic distribution of *hgcA* genes in Frozen Pond Fen in 2016 and their relationship with pore water %MeHg. **(A)** Normalized *hgcA* gene copies in shotgun metagenomes and the %MeHg decline with increased distance across the fen gradient. Pearson (R) and Spearman (ρ) correlation statistics are displayed. **(B)** Class level distribution of *hgcA* sequences from the shotgun metagenomes (left) and amplicon sequencing (right). **(C)** Family level distribution of *hgcA* reads from shotgun metagenomes and amplicon sequencing.

*hgcA* homologs were extracted from shotgun metagenomes and taxonomically classified across the gradient to assess the genetic potential for mercury methylation. In 2016, *hgcA* relative abundance was significantly negatively correlated with distance from FPF1 (Pearson’s *r* = −0.83 *p* = 0.042) and showed similar trends of %MeHg along the gradient (ρ = −0.77, *p* = 0.1) (Figure 2A).

Bacterial *hgcA* sequences were more abundant than archaeal sequences in the shotgun metagenomes from all sites (average bacterial abundance = 97 genes per metagenome; average archaeal abundance = 19.7 genes per metagenome), with *Desulfobacterota* (formerly *Deltaproteobacteria*) being the dominant phylum of *hgcA* sequences classified (Figure 2B). Sequences that had amino acid identity scores >60% were classified further to family level. In sites FPF1, FPF2, FPF4, and FPF5, >38% of *hgcA* sequences belonged to families that can participate in obligate or facultative syntrophic interactions including *Syntrophaceae, Desulfobacteraceae, Syntrophorhabdaceae, Syntrophomonadaceae, Methanoregulaceae, Methanospirillaceae, Methanocorpusculaceae*, and *Methanosarcinaceae* (Figure 2C). *hgcA* gene-homolog families belonging to the class *Clostridia* were in higher relative abundance in sites FPF2, FPF4, and FPF5 when compared to the nutrient rich site, FPF1 (Figure 2C). Site FPF6 had no *hgcA* sequences with identity scores >60% in 2016, so family data is displayed as unclassified (Figure 2C). *hgcA* diversity was highest in FPF1, followed by decreasing diversity along the gradient with a low at site FPF5 (Supplementary Table 3).

In comparison to the shotgun metagenomes, *hgcA* sequences obtained with high-throughput, primer-based amplicon sequencing aligned with only two Hg methylating taxa, *Desulfobacterota* and *Methanomicrobia* (Figure 2B, *Methanomicrobia* reads <1%). This is not surprising given the PCR primer bias toward the *Desulfobacterota* (Schaefer J. K. et al., 2014) and the abundance of these classes in the metagenome libraries. This PCR approach had clear limitations for identifying alternative groups of potential Hg methylators (e.g., *Clostridia*) and assessing the relative distribution of families within the *Desulfobacterota*. Two families of SRB, *Desulfobacteraceae* and *Desulfovibrionaceae*, known for robust Hg methylation activities (Gilmour et al., 2011), dominated the *hgcA* amplicon sequences in FPF1 despite their low abundance in the metagenome libraries. *Geobacteraceae hgcA* amplicon reads were detected in all FPF sites, albeit at low levels with a maximum proportion of 6% in FPF6 (Figure 2C), in agreement with the metagenome libraries.

In 2018, *hgcA* copy number from shotgun metagenomes was not significantly correlated with distance from FPF1, and a more even distribution of *hgcA* genes was observed along the gradient (Pearson’s *r* = −0.46, *p* = 0.36) when compared to 2016 (Supplementary Figure 6A). Average *hgcA* diversity was higher than in 2016 with lower variability (Shannon’s H 2016 = 2.12 ± 1.01, 2018 = 2.52 ± 0.49), however, this difference was not significant (Kruskal-Wallis, *p* = 0.44) (Supplementary Table 3). Similar to 2016, site FPF1 had the highest diversity of *hgcA* sequences in 2018 (Supplementary Table 3). *hgcA* sequences that aligned with organisms with syntrophic metabolism were highest in sites FPF1 and FPF2, whereas sequences most similar to those of the class *Clostridia* were found in highest relative abundance in FPF5 (Supplementary Figure 6C). All FPF sites in 2018 had *hgcA* sequences that aligned with metabolically diverse microbial families (Supplementary Figure 6C).

### Functional Gene Analysis

Results from the mercury methylating community were further validated by investigation of marker genes for microbial metabolism. In both years (2016 and 2018), *dsrB*, a marker for sulfate reduction, was highest in FPF1, coincident with the highest sulfate porewater concentrations (Supplementary Table 5). *dsrB* abundance tended to decline along the gradient, although the trend was weaker in 2018 (Pearson’s *r* = −0.59, *p* = 0.22) than in 2016 (Pearson’s *r* = −0.734, *p* = 0.097). The average *dsrB* gene counts were higher in 2018 (mean = 96.5) compared to 2016 (mean = 64.9) when sulfate concentrations in the groundwater inflows were also higher (Supplementary Table 2). A significant correlation between *hgcA* and *dsrB* was seen across the gradient in 2016 (Pearson’s *r* = 0.98, *p* < 0.001), but not in 2018. The marker gene for methanogenesis, *mcrA*, was detected in all sites and had no clear trend in either year (Supplementary Table 5).

The gene encoding for the mercuric reductase, *merA*, was detected along the gradient in both years (Supplementary Table 5). No trend in normalized abundance was observed along the gradient in either year. While *merA* encodes for one potential mechanism for Hg(0)_(aq)_ formation, no significant correlation was observed between *merA* abundance and Hg(0)_(aq)_, suggesting other mechanisms (Wiatrowski et al., 2006; Gregoire et al., 2018) may be important for the elevated levels of Hg(0) detected in the fen (Table 1; Poulin et al., 2019).

### Potential Mercury Methylation Rates

To assess the impact of the groundwater input on Hg methylation, potential Hg methylation rates by peat slurries were measured in sites FPF2 and FPF5 representing the nutrient rich and nutrient poor portions of the gradient, respectively. Treatments, designed to elucidate the contribution of specific microbial guilds to Hg methylation, included (1) sulfate addition, which can stimulate SRB, (2) molybdate addition, which inhibits SRB, (3) BES addition, which inhibits methanogenesis (Oremland and Capone, 1988), and (4) molybdate and BES which inhibit both SRB and methanogens.

**FIGURE 3:**
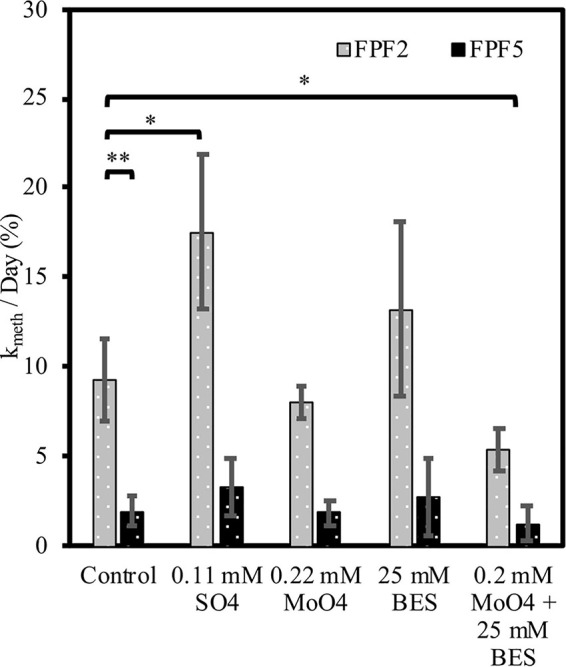
Mercury methylation rates (k_*meth*_) at sites FPF2 (gray bars) and FPF5 (black bars) in 2016. X-axis labels indicate treatment with stimulator or inhibitors of specific microbial guilds. Error bars represent standard deviations. * means *p* < 0.10, ** means *p* < 0.05.

Potential methylation rate constants in unamended incubations were nearly 5-fold higher (*p* = 0.013) in FPF2 than in FPF5 (9.3 and 2.0 k_*meth*_/day, respectively) (Figure 3) in 2016, corresponding to higher *hgcA* gene abundances and diversity in the former (Figure 2). Sulfate addition (∼0.11 mM) to the FPF2 slurry incubation increased k_*meth*_ 1.8-fold in 2016 (*p* = 0.09) when compared to the control slurry (Figure 3). This suggests that Hg methylation in the control slurries was potentially limited by sulfate availability and that the community was primed to utilize sulfate, consistent with the presence of SRB in 16S rRNA libraries (Figure 1) and *dsrB* genes in the shotgun metagenomes (Supplementary Table 5). However, k_*meth*_ in FPF2 was not significantly impacted by the addition of molybdate, suggesting SRB played a limited role in Hg methylation in the control incubation. While SRB were present and active (Figures 1, 2), the lack of significant reduction in k_*meth*_ by molybdate indicates that other microbial guilds substantially contributed to methylation to mask the impact of the inhibitor on k_*meth*_ (Figure 3). The specific inhibitor of methanogenesis, BES, had no significant effect on k_*meth*_, likewise ruling out methanogens as the major Hg methylating group in FPF2 (Figure 3). However, when molybdate and BES were added concurrently, a 42% decrease in k_*meth*_ was observed (2016 *p* = 0.076; not significant) (Figure 3), supporting a role for both SRB and methanogens in the methylation of mercury that was not detected when inhibitors were added individually.

In contrast, incubations from site FPF5 were not significantly impacted by sulfate or inhibitor (molybdate/BES) amendment in 2016. This indicates that methylation of Hg was likely carried out by a metabolic guild that was not impacted by BES or molybdate (Figure 3) such as IRB or fermenters. In fact, *Geobacteraceae hgcA* genes were detected in highest relative abundance in sites FPF3–FPF6, suggesting a potential role for IRB in Hg methylation down-gradient (Figure 2 and Table 1).

In 2018, k_*meth*_ was higher in both sites compared to 2016, and statistically similar (*p* = 0.82) in the FPF2 and FPF5 control incubations (Supplementary Figure 7). Similar to 2016, k_*meth*_ was 1.7-fold higher when sulfate was added. When added independent of one another, molybdate and BES had no significant impact on Hg methylation rates in FPF2, but concurrent addition of both inhibitors resulted in a 45% decrease in k_*meth*_ as seen in 2016 (Figure 3).

Contrary to 2016, Hg methylation in incubations from FPF5 in 2018 was significantly stimulated by the addition of either molybdate and BES when added alone, but not when added concurrently (Supplementary Figure 7). Sulfate addition resulted in a 2-fold increase in k_*meth*_, however, this was not significant (*p* = 0.25) (Supplementary Figure 7). These results suggest that SRB and methanogens were not the only methylating guilds in FPF5. Molybdate and BES may have stimulated guilds not impacted by the inhibitors by reducing competition for resources, one possible explanation for the increase in k_*meth*_. As in 2016, metabolic guilds not tested for could be primary Hg methylators in FPF5, including IRB and fermenters.

### Volatile Fatty Acid Accumulation in Peat Slurry Incubations

**FIGURE 4:**
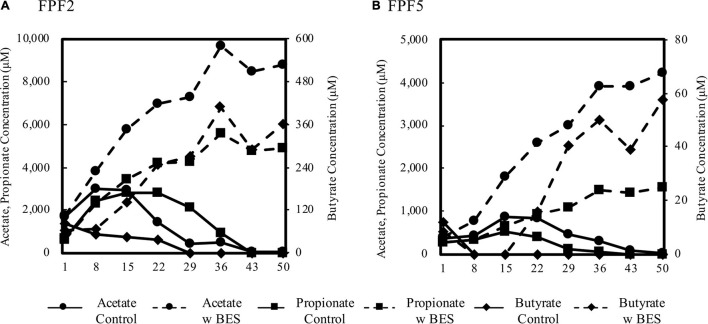
Concentration of acetate, propionate, and butyrate over 50 days in peat slurry incubations from FPF2 **(A)** and FPF5 **(B)**. Solid lines represent no-amendment controls and dashed lined represent incubations to which BES was added to inhibit methanogens. Acetate and propionate concentrations are displayed on the left y-axis and butyrate is displayed on the right. Note the difference in y-axis scales between subplots **(A)** and **(B)**.

The abundance of *hgcA* homologs closely related to syntrophs and the significant decrease in k_*meth*_ with concurrent addition of BES and molybdate indicated that syntrophic interactions may have contributed to Hg methylation in the fen. To further determine the role of syntrophy in the fen, we monitored VFAs in peat slurry incubations of 2016 samples with and without the methanogen inhibitor BES as an indication of syntrophic interactions between methanogens and VFA fermenters. After an initial pulse of VFAs from slurry preparation and organic matter decomposition, VFA concentrations declined in unamended control incubations (no BES) reaching below detection levels by day 43 (Figure 4 and Supplementary Figure 8). When methanogens were inhibited by the addition of BES, VFA concentrations (i.e., butyrate, propionate, and acetate) did not decline, but continued to accumulate until plateauing to a constant level after ∼36 days. By consuming the products of propionate and butyrate fermentation (acetate and H_2_), methanogens promote the fermentation of these VFAs. Propionate and butyrate are not directly utilized by methanogens (Evans et al., 2019), thus their accumulation in the presence of BES demonstrates the importance of syntrophic interactions between VFA oxidizers and methanogens in driving mineralization of organic matter when terminal electron acceptors are depleted.

Production and consumption of VFAs was faster in FPF2 than in FPF5, and BES treatment resulted in 2–6-fold higher concentrations of individual VFAs in FPF2 when compared to FPF5. Known syntrophic taxa, such as the *Syntrophaceae*, were higher in relative abundance nearer the groundwater source (FPF1 and FPF2) than further downgradient (FPF5), coincident with higher VFA accumulations following BES treatment (Supplementary Table 4).

Similar responses to BES treatment were observed in the other FPF sites, except for site FPF4, where VFAs accumulated in both control and treatment incubations (Supplementary Figure 8). Methanogenic taxa and *mcrA* genes were detected in FPF4 at abundances similar to the surrounding sites (Supplementary Table 5), and initial acetate concentrations in the FPF4 slurry were higher than all other sites, possibly disrupting the system. Collectively, the results across the fen suggest syntrophy is a major mineralization pathway of secondary fermentation products (i.e., VFAs) and support results of the community analysis and Hg methylation incubations.

## Discussion

We examined an Alaskan fen to evaluate the effects of groundwater nutrient inputs on soil microbiome structure and microbial Hg methylation. Our results (1) connect nutrient inputs in northern peatlands to increased diversity and abundance of Hg methylating communities and increased Hg methylation potentials, and (2) document the role of syntrophic interactions in Hg methylation. To our knowledge, this is the first study to pair activity measurements with *hgcA* sequencing through shotgun metagenomics to reveal the role of syntrophy in Hg methylation in the environment. Importantly, our results provide evidence for the involvement of syntrophy in Hg methylation in an ecosystem that is critical to the global Hg cycle due to vast stores of Hg with connections to aquatic food webs in the Arctic and subarctic (Dietz et al., 2013).

Nutrient inputs in Frozen Pond Fen impacted microbial community structure and Hg methylation rates. Microbial communities clearly responded to nutrient inputs in the Frozen Pond Fen (Table 1 and Supplementary Figure 4) as evidenced by a corresponding decline in community diversity away from the source (Supplementary Table 3). More importantly, the abundance of *hgcA* gene homologs in peat metagenomes declined significantly along the gradient, closely reflecting the decline in %MeHg (Figure 2A). These negative correlations with distance from the groundwater source suggest that nutrient inputs provide conditions that promote activity of Hg methylating microorganisms in the fen. Groundwater brings electron donors and acceptors (Table 1), supporting anaerobic respiration such as that carried out by SRBs. This was reflected in the community composition as SRB were more abundant nearest the groundwater source at FPF1 and FPF2 (Figure 2 and Supplementary Table 5). Despite no decrease in k_*meth*_ with molybdate addition in FPF2, sulfate addition significantly enhanced Hg methylation in FPF2 incubations (Figure 3). These results suggest that SRB were not directly contributing to Hg methylation in FPF2 but that the community was primed to utilize sulfate and that subsequent releases of sulfate into the fen could promote MeHg production by directly stimulating SRB Hg methylators or by stimulating non-methylating SRB that support the growth of Hg methylating syntrophs. Sulfate-reducing bacteria are well-known, efficient Hg methylators (Gilmour and Henry, 1992; Gilmour et al., 2013), and our results indicate that increases in warming-associated nutrient inputs (Reyes and Lougheed, 2015) may stimulate SRB, resulting in increased MeHg accumulation in these wetlands. While SRB may be a minor component of the total microbial community, their activity can contribute significantly to organic matter breakdown in fen systems even at low micromolar sulfate concentrations (Pester et al., 2010).

Our conclusion is supported by previous research in wetlands that has shown sites of intermediate trophic status, such as fens, have high net MeHg production. These intermediate sites have higher k_*meth*_ compared to ombrotrophic bogs (Tjerngren et al., 2012; Poulin et al., 2019; Schaefer J. K. et al., 2020), while having lower rates of demethylation compared to eutrophic swamps (Kronberg et al., 2012). Fens generally have higher pH and sulfate concentrations than bogs, and a link between those pore water geochemical parameters and %MeHg has been observed (Gordon et al., 2016). Furthermore, Hg methylation potential (k_*meth*_) in peatlands has been shown to be significantly correlated with a natural sulfur gradient and could be stimulated with experimental sulfur addition (Akerblom et al., 2020), in line with our results showing increased k_*meth*_ with sulfate addition in the Frozen Pond Fen. The results from the Frozen Pond Fen expand our understanding of Hg dynamics in peatlands by linking the genetic potential for Hg methylation with previously reported trends in Hg methylation potentials based on nutrient status.

When electron acceptors such as sulfate become depleted, syntrophy becomes an increasingly more important metabolic pathway. Here we show that that increase promotes Hg methylation. Molecular evidence and activity measurements in the Frozen Pond Fen incubations both provide evidence for Hg methylation through syntrophy. Many FPF metagenomic *hgcA* reads are closely related to those of organisms that can participate in facultative or obligate syntrophy in the nutrient rich sites, including methanogenic families as well as *Syntrophaceae*, *Desulfobacteraceae, Syntrophorhabdaceae*, *Syntrophomonadaceae, and Peptococcaceae* (Figure 2C). The 16S rRNA sequences validated these results, with a large relative abundance of *Syntrophaceae* and *Syntrophobacteraceae* in nutrient richer sites (Supplementary Table 4). The *Syntrophorhabdaceae, Syntrophomonadaceae*, as well as two genera in the *Syntrophaceae, Syntrophus, and Smithella*, are largely obligate syntrophs requiring an H_2_ scavenging partner (Kuever, 2014a, b). The *hgcA* amplicon sequencing showed high relative abundances of two SRB families that could participate in facultative syntrophic interactions; however, a clear discrepancy between sequencing methods was observed (Figure 2). Our results show that metagenomic sequencing may better describe Hg methylator diversity due to reduced bias compared to primer-based approaches as has been previously suggested (Christensen et al., 2019). Overwhelmingly, our results indicate that the genetic potential for Hg methylation by syntrophy is dominant in the Frozen Pond Fen, especially in nutrient rich sites (Figure 2).

The molecular results supporting syntrophy involvement in Hg methylation were validated by peat incubations in FPF2 and FPF5 of both Hg methylation potential and VFA dynamics (Figures 3, 4, respectively). The lack of inhibition of k_*meth*_ by molybdate suggests methylation by microbial guilds other than SRB (Compeau and Bartha, 1985), possibly stimulated by their inhibition. Several methanogen species have been shown to methylate (Yu et al., 2013; Gilmour et al., 2018), however, BES had no inhibitory effect on MeHg formation (Figure 3). Together, these observations indicate that several guilds, likely interacting with each other, contributed to methylation. When molybdate and BES were added concurrently, Hg methylation was significantly inhibited in FPF2 (Figure 3) suggesting that syntrophs, likely coupled to H_2_ and acetate utilizers, contribute to MeHg formation in the fen. Two genera in the *Syntrophaceae*, as well as the *Desulfobacteraceae* and some *Peptococcaceae* (Lee et al., 2009) are able to grow syntrophically or reduce sulfate when it becomes available (Muyzer and Stams, 2008), potentially explaining stimulation with sulfate while no inhibition with molybdate was observed. Likewise, our results also showed that the VFAs acetate, propionate, and butyrate built up in incubations treated with BES (Figure 4). Because propionate and butyrate cannot be utilized directly by methanogens (Evans et al., 2019), their accumulation after BES treatment suggests that methanogens were growing in syntrophy with VFA fermenters. Acetate accumulated in BES treated slurries by the first time point (*t* = 1 day), indicating that methanogenesis was inhibited (Figure 4) while propionate and butyrate accumulated after 15 days. Fermentation of VFAs will be greatly inhibited if the byproducts of fermentation, namely acetate and H_2_, are not consumed. The time lag in propionate and butyrate build up with BES treatment could therefore indicate that non-methanogenic H_2_ and acetate utilizing organisms support the growth of syntrophs in FPF under shorter time periods (>15 days).

Syntrophic metabolic niches have been studied in peat-forming wetlands previously (Schmidt et al., 2016). The contributions of syntrophic interactions to MeHg production by syntrophic interactions has been recently suggested (Bae et al., 2014; Hu et al., 2020; Schaefer J. K. et al., 2020), and *hgcA* genes and transcripts closely related to the syntrophic genus *Smithella* have been detected in permafrost impacted peatlands (McDaniel et al., 2020). In order to be active and contribute to MeHg production, syntrophs must metabolize with a partner microorganism that can utilize secondary fermentation end products such as H_2_ and acetate (Muyzer and Stams, 2008). Many methanogens and SRB can utilize acetate or H_2_, and SRB will outcompete methanogens when sulfate is available. In the absence of sulfate, SRB can also grow syntrophically with methanogens by fermenting volatile fatty acids (Muyzer and Stams, 2008). Yu et al. (2018) showed that these various syntrophic interactions may support methylation of Hg regardless of which syntrophic partner has methylating capabilities. Correspondingly, we observed significant reduction in k_*meth*_ in FPF peat only when both major H_2_ and acetate utilizing guilds were inhibited by BES and molybdate. As contemporary sequencing methods continue to identify the full extent of *hgcA* presence and diversity, it is important to consider the contribution of newly identified guilds to MeHg production in the environment. By pairing activity measurements with sequence-based approaches, our results robustly indicate Hg methylation by microorganisms growing in syntrophy in the environment.

We did not directly test the role of IRB or fermenters in Hg methylation through our inhibitor incubations. However, when both BES and molybdate were added to peat incubations, MeHg was still formed in both sites in both years (Figure 3). This MeHg formation is not attributed to SRB, methanogens, or major syntrophic pathways, suggesting a role for other microbial metabolic guilds. 16S rRNA sequencing and metagenomes *hgcA* gene homologs showed an abundance of sequences associated with the IRB family, *Geobacteraceae*, as well as the class *Clostridia* which includes Hg methylating strains that are metabolically diverse, including SRB, IRB, fermenters, dehalorespirers, and microorganisms that can participate in syntrophy (Figure 2). Species in *Geobacteraceae* have been shown to be strong methylators in pure culture (Kerin et al., 2006; Schaefer and Morel, 2009) and have been associated with environmental MeHg production (Bravo et al., 2018). Hg methylators within the class *Clostridia* grown in pure culture have lower methylation rates than the phylum *Desulfobacterota*, in general (Gilmour et al., 2013). Some *hgcA* sequences in the metagenomes were also related to *Dehalococcoidia* and *Bacteroidia*, two classes for which Hg methylation potentials are unknown.

Comparing the results of our 2016 sampling with those obtained for the same locations in 2018 supports our conclusion. Trends in k_*meth*_ and community characteristics were less defined in 2018 (Supplementary Figure 7), corresponding to a dampened geochemical gradient (Supplementary Figure 4). Increased precipitation could have diluted nutrients in FPF in 2016 (Siegel and Glaser, 1987), and may have resulted in a higher water table which could inhibit microbial activity and impact MeHg concentrations (Haynes et al., 2017). Unlike in 2016, k_*meth*_ in FPF5 were similar to those observed in FPF2 (Supplementary Figure 7) and the decline in *hgcA* gene copy number along the gradient was much less pronounced (Supplementary Figure 6). Taxonomic classification of *hgcA* reads in 2018 revealed abundant SRB, syntrophic, and methanogenic families (Supplementary Figure 6), a finding that was further validated by the 16S rRNA results (Figure 1). Similar to results from site FPF2 in 2016, the concurrent addition of molybdate and BES to peat incubations in 2018 led to a significant decline in k_*meth*_ in both sites, indicating that syntrophy contributed to Hg methylation (Supplementary Figure 7).

In peatland ecosystems underlain by permafrost, rapid thawing as a result of increasing air temperatures is causing numerous changes including expansion of the active layer (Akerman and Johansson, 2008), release of previously sequestered nutrients into groundwater or surface water (Reyes and Lougheed, 2015), and changes in hydrology. While we did not determine the hydrology across FPF, pore water geochemical factors were significantly correlated with distance from the edge of the fen where a groundwater input was identified (Table 1). The impacts of warming-associated changes on microbial activities (Jansson and Tas, 2014) and supply of nutrients (Ma et al., 2019) are profound. Collectively, our results show that Frozen Pond Fen is an ecosystem in transition, biogeochemically responding to environmental changes. As the Arctic and subarctic are altered in response to climate change, conditions in ombrotrophic peatlands may shift to favor robust Hg methylating communities. Paired with potential Hg release from permafrost (Rydberg et al., 2010; Mu et al., 2020; Schaefer K. et al., 2020), these changes point to a potential for increased MeHg production and release from northern wetlands.

## Data Availability Statement

The datasets presented in this study can be found in online repositories. The names of the repository/repositories and accession number(s) can be found below: https://arcticdata.io/catalog/view/; doi: 10.18739/A2X921J5D; https://genome.jgi.doe.gov/portal/EffofApeatlands/EffofApeatlands.info.html, 504041.

## Author Contributions

TB, MH, JS, DK, BP, and SR: conception and design of experiments. SR, BP, ZB, XL, LZ, DK, MH, JS, and TB: collection, analysis, and interpretation of all data. SR: writing of the manuscript. BP, ZB, LZ, JS, and TB: revision of the manuscript. All authors contributed to the manuscript and have approved the submitted version.

## Conflict of Interest

The authors declare that the research was conducted in the absence of any commercial or financial relationships that could be construed as a potential conflict of interest.

## Publisher’s Note

All claims expressed in this article are solely those of the authors and do not necessarily represent those of their affiliated organizations, or those of the publisher, the editors and the reviewers. Any product that may be evaluated in this article, or claim that may be made by its manufacturer, is not guaranteed or endorsed by the publisher.

## Footnotes

1
https://genome.jgi.doe.gov/portal/

2
hmmer.org

3
https://img.jgi.doe.gov/
