# Supplementary material for: Nutrient Inputs Stimulate Mercury Methylation by Syntrophs in a Subarctic Peatland
Source: Front Microbiol. 2021 Oct 4;12:741523. doi: 10.3389/fmicb.2021.741523 (PMC8524442; doi:10.3389/fmicb.2021.741523)
Supplement: Supplementary file 1 [file Data_Sheet_1.DOCX]

**Supplemental Methods**

*Enriched Mercury Stable Isotope Analysis*

Digestion and analysis of Hg from the 2018 samples was performed at the University of Connecticut in the laboratory of Prof. Mason using established methodology. Measurements included MeHg and THg, where the isotopic composition of Hg was differentiated through gas chromatography inductively coupled plasma mass spectrometry (GC-ICPMS). To determine the isotopic composition of MeHg, ~2g of dried peat was weighed into 50 mL Falcon tubes. Internal standard of MeHg enriched with Me^198^Hg (isotopic composition: 93.53%), in the amount of either 0.9 ng or 6.1 ng (depending on background levels of previously measured THg in all samples), was added to each tube and equilibrated for approximately 24 hours. Digestion relied on the addition of 1M cupric sulfate, 18% potassium bromide solution in 5% H_2_SO_4_ (vol./vol. prepared from 18.4M) and dichloromethane (DCM; 10 mL) into the tubes with peat samples, following the incubation for an hour on a shaker table (Lambertsson et al., 2001). After this step, vials were centrifuged to separate solids from the liquid and as much of the DCM layer was removed and transferred into pre-weight 40 mL amber glass vials using Hamilton syringe while avoiding withdrawal of solid material. The exact amount of DCM that was removed from each Falcon tube was determined by weight. Amber vials containing DCM, deionized water and boiling chips were heated (60˚C) and purged with N_2_ for 30 minutes to remove all the DCM leaving aqueous solution of MeHg behind. After purging was completed weights of vials were checked to account for any losses of water during the heating and purging time. At this point solutions were spiked with 4.2M acetate buffer, and sodium tetratethylborate (NaBET_4_) solution to ethylate MeHg at least 20 minutes prior to analysis. Samples were analyzed using coupled system of automatic MeHg analyzer Tekran 2700 and ICP-MS (Perkin Elmer Elan DRC II). Data was post processed mathematically to deconvolute the signals attributable to the most prominent isotopes of Hg as expected in the MeHg extract. The calculation relied on the known amount of the internal standard that was added to each vial.

To enable the calculation of methylated ^200^Hg(II) in each peat sample, knowledge of total amount of this Hg isotope was required even though each peat vial received the same amount of spike. Therefore, the total amount of Hg (THg) in peat samples was determined based on addition of known amount of internal standard i.e., HgCl_2_ enriched with ^198^Hg (isotopic abundance: 93.53%) to peat samples that ranged in mass from 0.05 to 0.30 g dry wt. One sample was weighed in triplicate to determined analytical error. Samples were digested following the EPA 1631 method (Digestion I - for samples with high organic matter content) using 70:30 vol./vol. mixture of concentrated nitric and sulfuric acids for 24 hours, following with dilution by 0.2 N bromine monochloride (BrCl) to oxidize any remaining organic matter. An aliquot of the digest was mixed with deionized water, the remaining halogens were neutralized by addition of hydroxylamine, and inorganic Hg was reduced to its elemental form by addition of stannous chloride (SnCl_2_) 20 minutes prior to analysis. Analysis was performed using Total Mercury Analyzer Tekran 2600 coupled with the ICP-MS. As for MeHg, isotopic composition was determined based on the known amount of isotope that was added as internal standard. Isotopic signals from the MeHg and THg analyses were mathematically deconvoluted (Qvarnstrom and Frech, 2002) after accounting for instrumental mass bias based on triplicate analyses of ambient Hg standards. Isotopic signals contributing to less than 5% were considered to be below the detection limit and not included in final data analysis.
