# Supplementary material for: Nutrient Inputs Stimulate Mercury Methylation by Syntrophs in a Subarctic Peatland
Source: Front Microbiol. 2021 Oct 4;12:741523. doi: 10.3389/fmicb.2021.741523 (PMC8524442; doi:10.3389/fmicb.2021.741523)
Supplement: Supplementary file 2 [file Data_Sheet_2.docx]

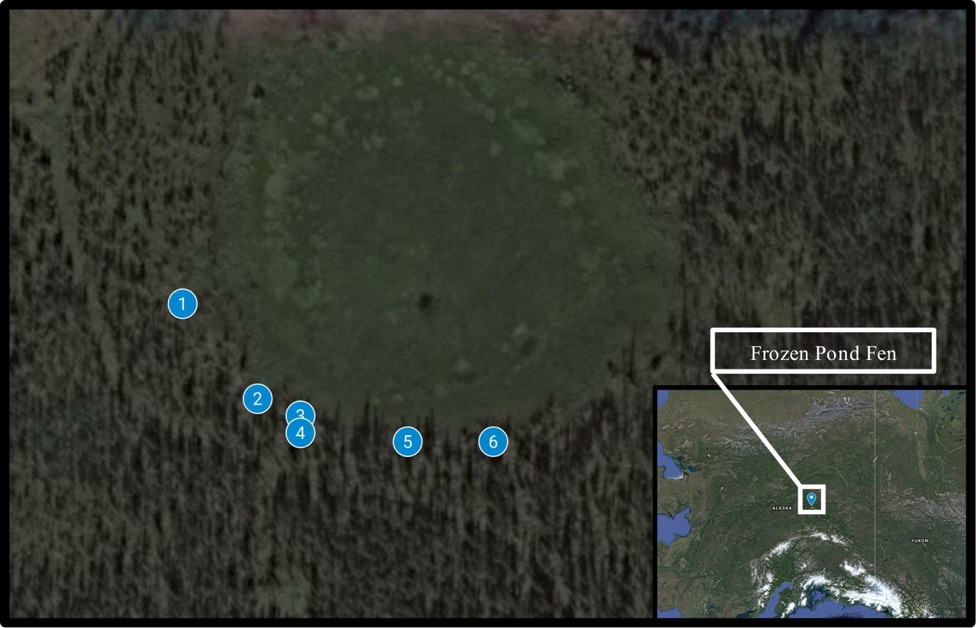


Figure S1: Frozen Pond Fen site map for sampling locations in 2016 and 2018. Sites are numbered according to FPF identification numbers. A groundwater input was identified near site FPF1.

Table S1: Metagenome read count summary for 2016 and 2018.

| Site | Gene Count 2016 | Aligned read count 2016 | Gene Count 2018 | Aligned read count 2018 |
| --- | --- | --- | --- | --- |
| FPF1 | 1564342 | 29133061 | 2070043 | 42773882 |
| FPF2 | 1069376 | 19141542 | 2393715 | 50195544 |
| FPF3 | 1701151 | 31966741 | 1090012 | 18630777 |
| FPF4 | 1955465 | 34482196 | 1482263 | 32595569 |
| FPF5 | 1850911 | 37290934 | 1377438 | 24781900 |
| FPF6 | 1177841 | 25972965 | 3044289 | 59616463 |

Table S2: Site location and pore water geochemistry from 2018. All values below detection are denoted as less than the detection limit.

| **Site** | **FPF1** | **FPF2** | **FPF3** | **FPF4** | **FPF5** | **FPF6** |
| --- | --- | --- | --- | --- | --- | --- |
| Latitude | 64.914417 | 64.914233 | 64.9142 | 64.914167 | 64.91415 | 64.91415 |
| Longitude | -147.83545 | -147.8351 | -147.8349 | -147.8349 | -147.8344 | -147.834 |
| Distance from FPF1 (m) | 0 | 26.8 | 35.4 | 39.1 | 57 | 73.1 |
| Ca^2+^ (mmol/L) | 1.65 | 0.65 | 0.37 | 0.48 | 0.22 | BD^1^ |
| pH | 6.67 | 6.35 | 6.27 | 6.19 | 6.16 | 5.86 |
| DOC (mg C/L) | 197.2 | 89.2 | 55.8 | 84.1 | 36.1 | 15.5 |
| SO_4_^2-^ (μmol/L) | 184 | BD | BD | BD | BD | BD |
| Propionate (mg/L) | 0.13 | 0.10 | 0.16 | 0.08 | 0.20 | 0.08 |
| Acetate (mg/L) | BD | 2.79 | BD | BD | 3.17 | BD |

^1^BD, below detection. Detection limits were 0.12 mmol/L Ca^2+^ (Poulin et al, 2019), 1.2 µmol/L sulfate and 1.5 mg/L acetate.


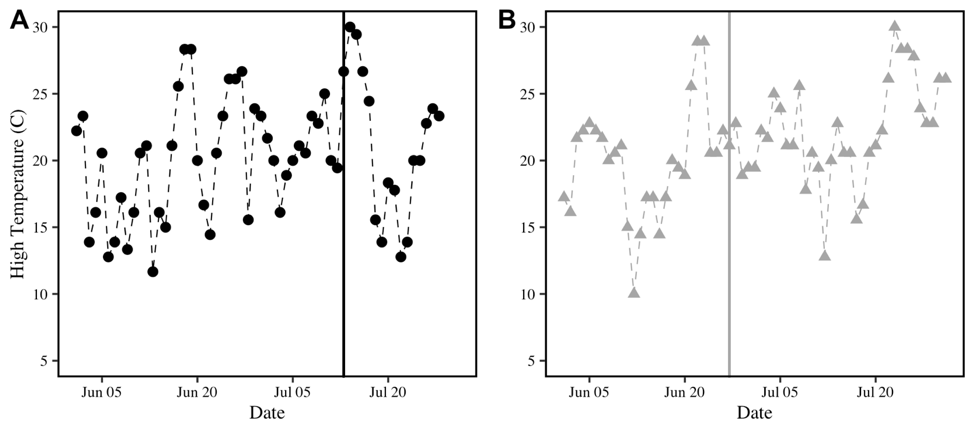


Figure S2: Daily high temperature measured at Goldstream Creek, near Fairbanks, AK. Data from 2016 (A) and 2018 (B) are displayed. Sampling date is denoted by the vertical line in each subplot. Data collected from NOAA National Centers for Environmental Information station USC00503368.


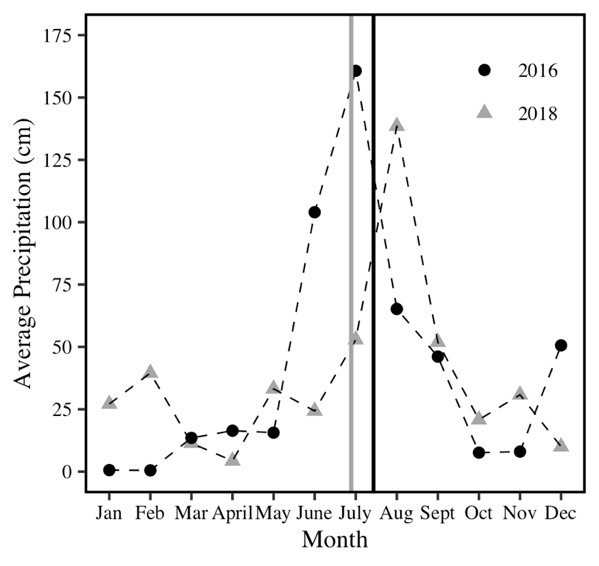


Figure S3: Average monthly precipitation at Goldstream Creek, near Fairbanks, AK. Averages from 2016 (black circles) and 2018 (gray triangles) are presented. Sampling date is denoted by the vertical lines. Average precipitation preceding sample collection was higher in 2016 compared to 2018. Data collected from NOAA National Centers for Environmental Information station USC00503368.


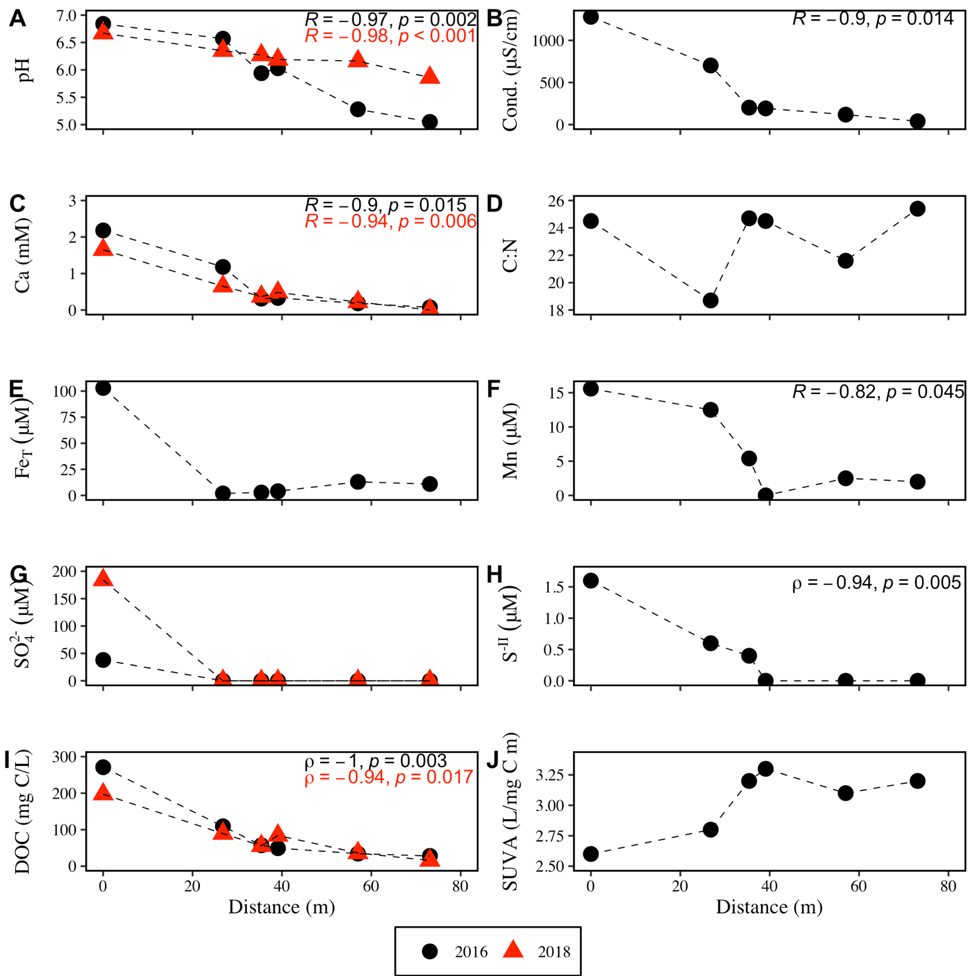


Figure S4: Pore water geochemical profiles across the gradient in 2016 (circles) and 2018 (triangles). Pearson’s (R) and Spearman’s (ρ) correlation statistics are displayed for factors significantly correlated with distance.


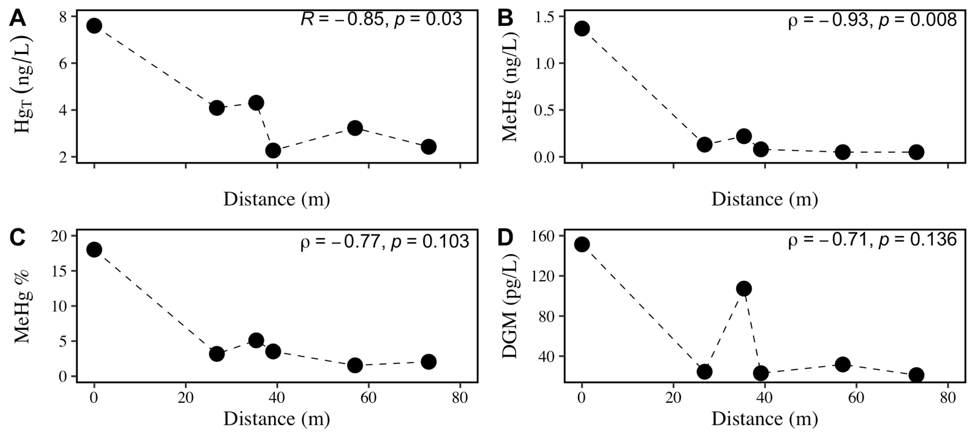


Figure S5: Mercury species measured in site pore water in 2016. Pearson’s (R) and Spearman’s (ρ) correlation statistics are displayed for factors correlated with distance from FPF1.

Table S3: Alpha diversity (Shannon Index) based on 16S rRNA amplicon reads and *hgcA* reads classified from the shotgun metagenomes.

| Site | Shannon Diversity Total Community 2016 (RNA) | Shannon Diversity Total Community 2018 (RNA) | Shannon Diversity 2016 metagenome *hgcA* | Shannon Diversity 2018 metagenome *hgcA* |
| --- | --- | --- | --- | --- |
| FPF1 | 7.11 | 9.16 | 3.223 | 3.087 |
| FPF2 | 5.61 | 9.41 | 2.917 | 2.851 |
| FPF3 | 5.91 | 9.84 | 0.693 | 1.667 |
| FPF4 | 6.88 | 9.15 | 2.476 | 2.614 |
| FPF5 | 4.57 | 9.94 | 2.303 | 2.425 |
| FPF6 | 5.39 | 9.83 | 1.099 | 2.451 |


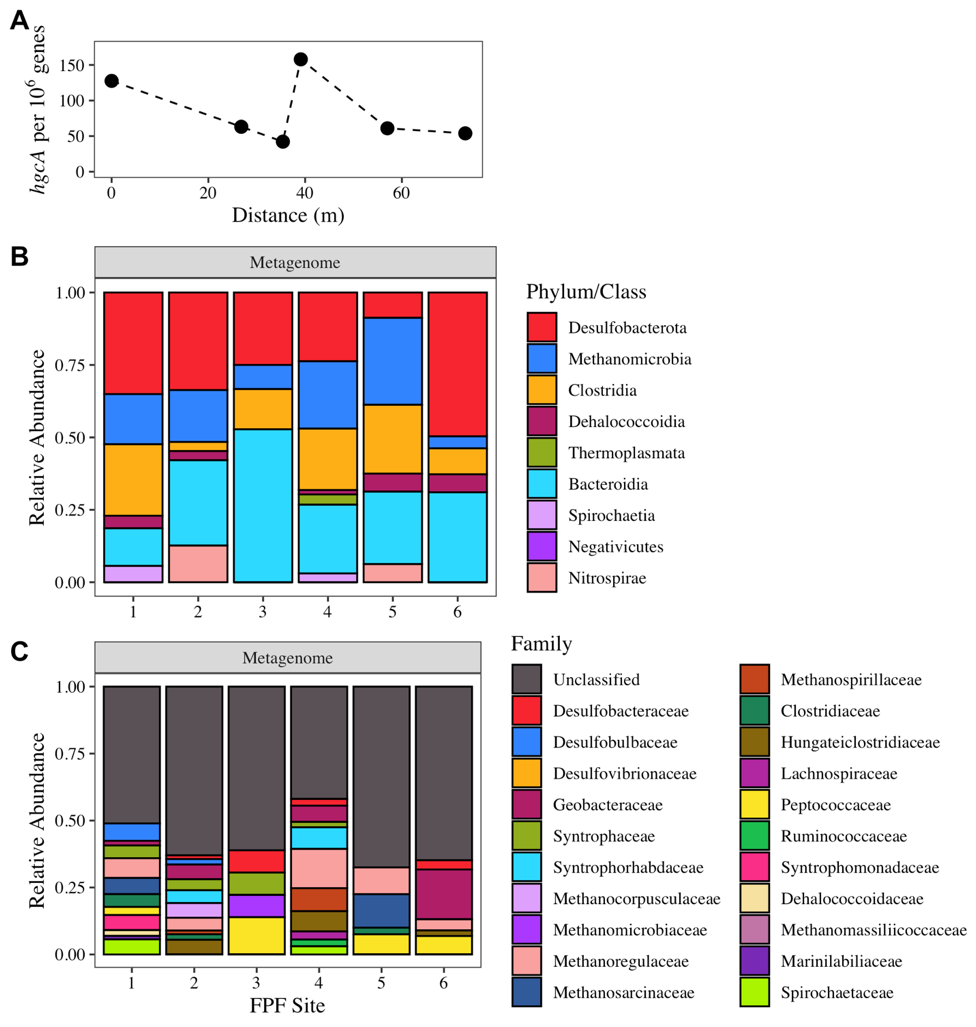


Figure S6: *hgcA* abundance and taxonomic distribution across the Frozen Pond Fen gradient in 2018. A) Abundance of *hgcA* homologs from shotgun metagenomes across the six FPF sites. B) Phylum/Class level and C) family level taxonomic distribution of *hgcA* genes in FPF. Relative abundances are presented and homologs that could not be classified to family level are presented as “Unclassified”.

Table S5: Marker gene counts per 1 million genes for methanogenesis (*mcrA*), sulfate reduction (*dsrB*), and mercury reduction (*merA*).

| Site | *mcrA* 2016 | *mcrA* 2018 | *dsrB* 2016 | *dsrB* 2018 | *merA* 2016 | *merA* 2018 |
| --- | --- | --- | --- | --- | --- | --- |
| FPF1 | 71.6 | 81.6 | 159.2 | 196.6 | 20.5 | 17.4 |
| FPF2 | 204.8 | 37.2 | 97.3 | 91.9 | 28.1 | 9.2 |
| FPF3 | 12.3 | 39.4 | 4.7 | 56.9 | 0.0 | 6.4 |
| FPF4 | 80.3 | 152.5 | 43.0 | 49.9 | 11.3 | 50.6 |
| FPF5 | 102.7 | 108.2 | 46.5 | 161.2 | 22.7 | 2.9 |
| FPF6 | 88.3 | 35.5 | 39.1 | 22.7 | 9.3 | 15.8 |


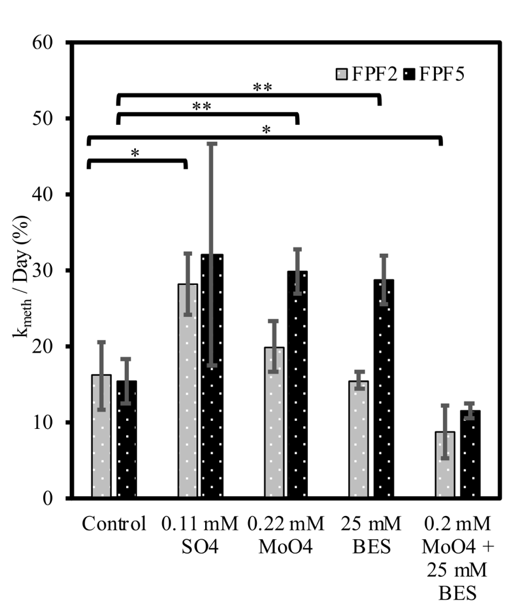


Figure S7: Mercury methylation rate constant (k_meth_) at sites FPF2 and FPF5 in 2018. X-axis labels indicate treatment with a stimulator or inhibitors of specific microbial guilds. Error bars represent standard deviations. * means p < 0.10; ** means p < 0.05.


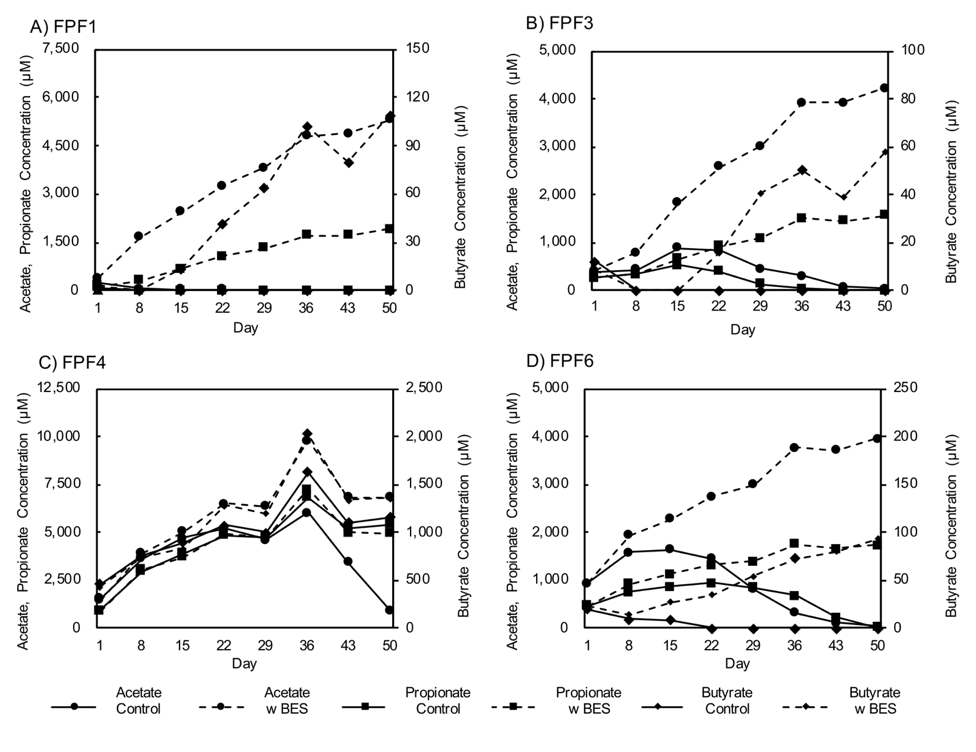


Figure S8: Concentration of acetate, propionate and butyrate over 50 days in peat slurry incubations from FPF1 (A), FPF3 (B), FPF4 (C), and FPF6 (D). Solid lines represent no-amendment controls and dashed lined represent incubations to which BES was added to inhibit methanogens. Acetate and propionate concentrations are displayed on the left y-axis and butyrate is displayed on the right. Note the difference in y-axis scales between the sites
